# Diabetic Immunotherapy Advances with BCG: Metabolic and Immune Reprogramming

**DOI:** 10.3390/cells15171604

**Published:** 2026-09-03

**Authors:** Denise L. Faustman, Shiho Hashiguchi, Miriam Davis, Willem Kuhtreiber

**Affiliations:** Immunobiology Laboratory, Massachusetts General Hospital and Harvard Medical School, Boston, MA 02129, USA

**Keywords:** aerobic glycolysis, metabolism, BCG, immunotherapy, autoimmunity, diabetes, LADA, Tregs

## Abstract

This review describes the cellular and molecular mechanisms underlying multi-dose bacille Calmette-Guérin (BCG) as an investigational immunotherapy for glycemic improvement in autoimmune diabetes. The BCG data is most often human clinical trial data over the last 15 years paired with mechanistic insights. BCG as a vaccine has an excellent safety record with a century-long safety record in tuberculosis prevention worldwide and, for 36 years, has been FDA-approved at higher doses for the treatment of bladder cancer. Our group and others have conducted multiple clinical trials evaluating multi-dose BCG immunotherapy in adults with the two forms of longstanding autoimmune diabetes. In type 1 diabetes (T1D), clinical trials of multi-dose BCG have shown stable repeated reductions in the FDA-recommended biomarker glycated hemoglobin (HbA1c) to near-normal levels (vs placebo) for at least 8 years. This improvement paradoxically occurs without any recovery of pancreatic function from its negligible baseline level. Evidence from long-term trials, together with companion global mechanistic studies, indicates that glycemic improvement is associated with BCG-induced correction of aerobic glycolysis defects in lymphoid and myeloid cells, an outcome also observed in the granuloma of BCG’s close relative, tuberculosis. Restoration of energy metabolism to aerobic glycolysis in diabetic lymphoid cells increases glucose utilization, thereby enhancing systemic glucose uptake from the bloodstream, as demonstrated by longitudinal PET/CT imaging. In contrast, latent autoimmune diabetes in adults (LADA), a form of slow autoimmune diabetes that occurs late into adulthood, has minimal or lacks the clinically significant aerobic glycolysis defect. In LADA with multi-dose BCG immunotherapy glycemic improvement in BCG-treated patients does not occur commonly, based on 8 years of data. LADA nevertheless displays other favorable immune and clinical responses suggestive of BCG-induced regulatory T cell (Treg) induction. In LADA, multi-dose BCG decreases inflammation, thus halting pancreatic autoimmunity with insulin rescue and also halts inflammatory driven insulin resistance. BCG’s consistent clinical benefits in autoimmune diabetes coincide with the gradual pace of upstream epigenetic reprogramming of genes involved in metabolic and T cell pathways without changing genotype. Understanding the mechanistic chain linking BCG-induced epigenetic changes to clinical efficacy may offer insights into the treatment and potential prevention of autoimmune diabetes.

## 1. Introduction to BCG as Immunotherapy

The bacille Calmette-Guérin (BCG) vaccine may be old, but it is not old-fashioned. While used globally since 1923 for tuberculosis (TB) prevention, it has versatile contemporary applications: as an FDA-approved immunotherapy for non-muscle-invasive bladder cancer and preventive intervention after high-risk TB exposure [1]. BCG is also an investigational intervention for diverse conditions, known as “off-target” effects, including multiple sclerosis [2,3], eczema and allergies in children [4], COVID and other infectious diseases [5,6,7,8], Alzheimer’s disease [9,10,11,12,13,14,15,16,17,18,19,20], and, the focus of this review, autoimmune diabetes (type 1 diabetes [T1D] and latent autoimmune diabetes of adults [LADA].

**Figure 1:**
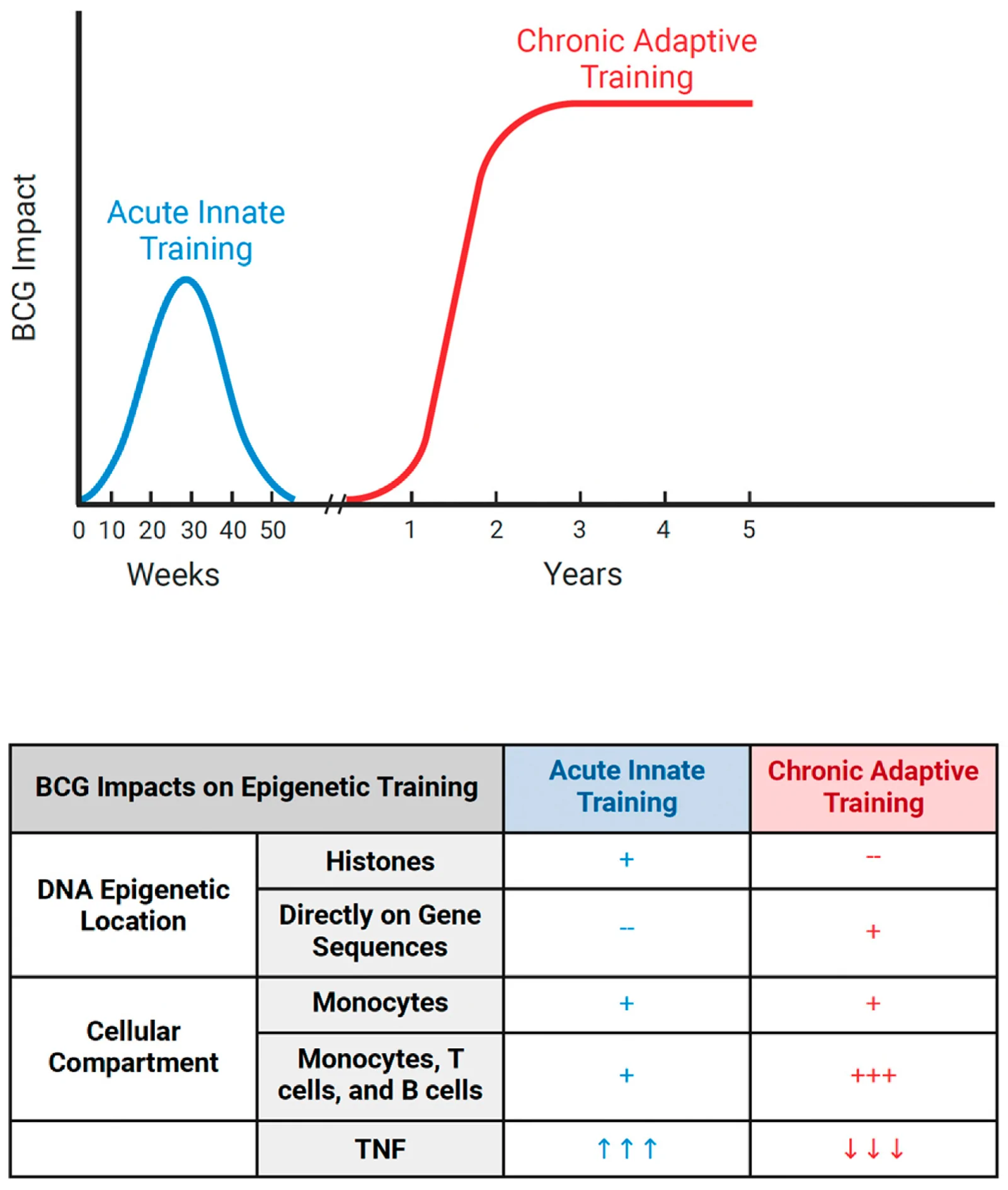
Time sequence and mechanism of methylation changes in acute innate training versus chronic adaptive training. Figure used a license from BioRender.

Contrary to most other immunotherapies for autoimmune diabetes, which are immunosuppressants—and thereby carry significant adverse effects—BCG has a strong safety record [21] and is the first immunostimulant under study for multiple autoimmune diseases such as autoimmune multiple sclerosis [22]. Its immunostimulant properties include cytokine release by activated innate immune cells, enhancement of antigen presentation, and induction of acute trained immunity (a form of “memory” by innate immune cells) [23] (Figure 1). In innate trained immunity, innate immune cells, once infected by BCG, develop memory-like characteristics: they show enhanced cytokine release upon restimulation by usually similar exposures and this is driven by histone changes from BCG induction of methylation alterations [23]. As will be discussed later in this review, the acute innate immune training is followed by chronic adaptive immune training in all cellular compartments of the immune system (T cells, B cells, monocytes) and this slower process does not involve histones but the actual epigenetic remodeling of direct gene pathways such as genes within the T-cell receptor, activation of suppressor Treg cells and rewiring of metabolic pathways involved in glucose utilization such as aerobic glycolysis [24,25,26,27,28,29,30,31,32] (Figure 1). BCG is a non-pathogenic strain of *Mycobacterium bovis*, the causative agent in bovine TB that does not infect humans. *Mycobacterium bovis* works as a tuberculosis vaccine because it resembles *Mycobacterium tuberculosis*, the causative agent of human TB. It has similarly been observed that the similar *Mycobacterium tuberculosis* strain also uses epigenetics for long-term survival in hosts such as the induction of Treg cells showing the overlap of these two ancient organisms for host interactions.

The ability of BCG to use epigenetics in human and animal models, and in cell culture, is well documented by an abundant literature of over 8000 papers in the last 10 years and is commonly referred to as innate immune training [33,34,35,36,37,38,39,40,41,42,43,44,45,46,47,48,49,50,51,52]. Similarly, the ability of BCG to model the adaptive immune system through epigenetics, a slower immune response also observed in vivo, is also a deeply studied topic within the scientific literature referred to as adaptive immune training [53,54,55,56,57,58,59,60,61,62,63]. These two mechanisms by which BCG molds the immune system in this review will only be referenced in the context of human studies in diabetes, wherein in some cases underlying abnormalities in diabetes will be highlighted to allow the BCG-induced epigenetic modeling to confer desirable therapeutic outcomes (Figure 1).

The goal of this review is to examine the cellular and molecular mechanisms by which BCG shows glycemic and/or disease-modifying benefits for T1D or LADA, the only two forms of autoimmune diabetes. We describe the evidence of BCG’s effects on immunometabolism and Treg activation, followed by the upstream role of epigenetic drivers. Epigenetically driven effects occur acutely and also unfold gradually over a 1–3-year period that coincides with the delayed onset of clinical outcomes. Finally, we consider how these mechanistic insights inform ongoing efforts in treatment and prevention of autoimmune diseases, as well as insights into the benefits of BCG immunotherapy in other forms of inflammatory disease such as Alzheimer’s disease.

## 2. BCG Clinical Trials Show Multiple Benefits for Autoimmune Diabetes

**Figure 2:**
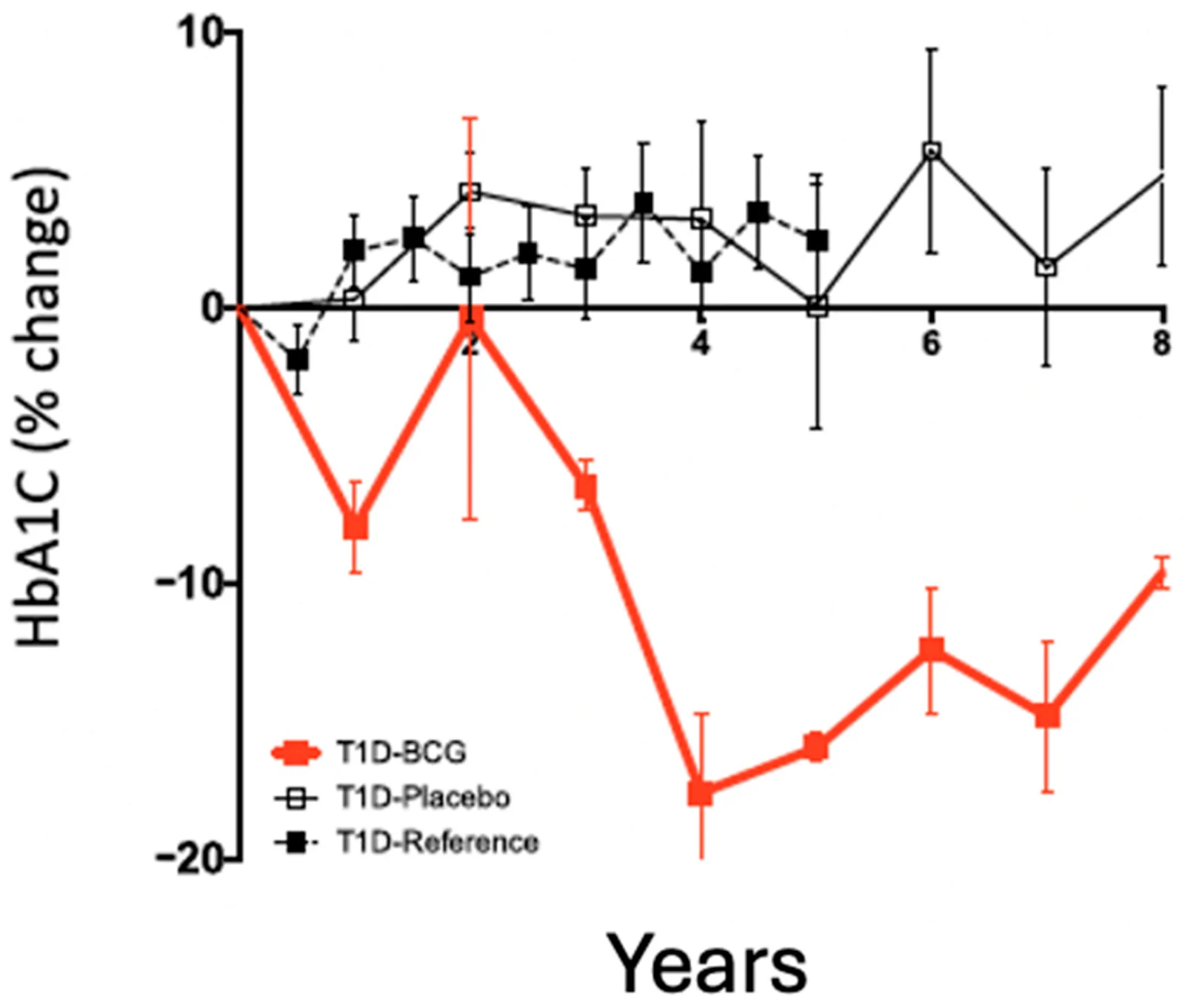
Long-term improvement of glycemic control in T1Ds after BCG treatment. Glucose control was tracked through measurements of HbA1c. HbA1c levels in the control T1D groups (both the saline-treated placebo group and the simultaneously matched reference group) remained unchanged over the 8-year observation period (placebo) or 5-year observation period (untreated reference group) as measured by a % change in HbA1c values [64].

We have completed five human clinical trials of multi-dose BCG immunotherapy for glycemic improvement in adults with longstanding T1D (Table 1) [28,64,65,66]. Two large double-blinded clinical trials are ongoing in pediatric diabetic subjects with blood sugar outcomes. One of those trials (Phase 2, 5–8 years) added a second study population with latent autoimmune diabetes of adults (LADA), defined by onset age ≥ 21 years. In these studies LADA was also identified by the presence of autoantibodies, most often GAD; a slow decline in pancreatic C-peptide secretion; and older age of onset. T1D is defined by onset age < 21 years. Two of the five trials were open-label, prospective studies [24,30]. Across the five trials, disease duration at enrollment ranged from >1 year to adulthood. Four trials found that BCG significantly improves glycemic control in T1D (Figure 2). One counterexample is the first trial (Phase 1A) which had, in retrospect, too short a follow-up period (20 weeks). The other counterexample was the LADA Phase 2, 8-year trial. LADA did not show glycemic improvement with 5 years of observations but did prevent insulin resistance and halted and restored, in part, pancreatic insulin secretion, confirming the removal of the autoimmunity. Both of these important clinical observations probably relate to the BCG-induced Treg induction removing inflammation. The failure of LADA to show glycemic improvement over 5 years supports our body of evidence regarding BCG’s mechanism of action includes correction of underlying metabolic defects in sugar metabolism if present at trial enrollment (see later section). Including diabetic trials with outcomes related to other off-target effects of BCG, such as protection from COVID-19, protection from all infectious diseases, protection from Alzheimer’s disease, a total of over 1140 consented type 1 diabetic subjects have been studied.

More specifically, T1Ds who received up to six doses of BCG immunotherapy were found after 2–3 years to have stably lower glycated hemoglobin (HbA1c) levels by approximately 7–15% (vs. placebo or baseline) up to the end of follow-up 8 years later [24,28,30,65,66]. The magnitude of improvement was clinically meaningful in terms of lower risk of microvascular complications, according to a well-respected study [67]. Also this study showed for the first time that BCG-treated long-term diabetics had long-term restoration of normoglycemia with blood sugars in the normal range from 70 to 99%, not in the hyperglycemia range. In our Phase 2 trial we found that HbA1c decline over 5 years was greatest, at almost 19%, among adult participants with the earliest age of onset (<12 years old), a finding that correlates with the magnitude of the aerobic glycolysis defect [65,66]. Unlike insulin, even when finely controlled with novel CGM and insulin pump technology, BCG does not induce hypoglycemia and shows much tighter glycemic control than standard of care with insulin pumps or continuous glucose monitoring (CGM) devices [65,66]. Although not a primary outcome of our studies, at year 8, 41% of our long-term type 1 diabetic subjects had HbA1c values that are in the normal range (less than 5.8 mg%) with no increased incidence of hypoglycemia.

Multi-dose BCG was found safe across all the trials, exhibiting no treatment-related moderate or serious systemic adverse events. The clinical trial evidence is supported by several retrospective or ecological studies showing that prior neonatal BCG for tuberculosis prevention was associated, years later, with improved glycemic control in T1D [68] or reduced incidence of T1D [69,70]. Prior high-dose BCG instillation for bladder cancer therapy was associated with later HbA1c improvement in T1D, but not type 2 diabetics [69].

LADA Results. The single BCG clinical trial with ample follow-up (5–8 years) that studied LADA (onset age ≥ 21 years) found no HbA1c improvement over this time frame. Nevertheless, BCG did have other clinically meaningful benefits for LADA; namely, prevention of insulin resistance, cessation of pancreatic islet loss (measured by C-peptide), and reduction in active autoimmunity [65,66]. These findings are mechanistically important in understanding BCG’s effects on immunometabolism and on Treg cells, both discussed subsequently.

## 3. Unusual Temporal and Clinical Characteristics of BCG Response

Our intensive study of BCG’s molecular and cellular mechanisms was launched by at least three unusual or unexpected characteristics encountered in clinical trials. The first is a delay of 2–3 years before the onset of glycemic improvement. In fact, we prematurely concluded, after the first trial of 20 weeks (Phase 1A RCT), that BCG was associated with no glycemic benefit, despite favorable biomarker results [64]. A few years later, we learned of a multiple sclerosis trial employing BCG as immunotherapy, showing clinical benefit after a 3–5 year delay [71]. We consequently then performed a Phase 1B trial with extended follow-up to year 8 [28]. Phase 1B results indicated that the extension was worthwhile: we succeeded by year 4 in showing, through serial blood sampling, a cumulative HbA1c decline from baseline of 18%. Many immunotherapies, with limited doses, take years to show benefit [72], including immune reconstitution therapies, adoptive cell therapies, immune checkpoint inhibitors, and gene therapies, not to mention other vaccines.

Another characteristic is the persistence of glycemic improvement years after limited dosing. Two RCTs [28,65,66] showed that BCG benefits lasted a minimum of 8 years. This characteristic is supported by subsequent mechanistic evidence, described in the next section, and also is supported by the RCT evidence regarding benefits of neonatal BCG. A striking example is an RCT of neonatal BCG for tuberculosis prevention: benefits were still measurable 60 years later [73]. At that late follow-up, the authors reported “only slight but not statistically significant waning of the efficacy.” There are no other RCTs of BCG for T1D treatment, much less observational studies reporting on the Tokyo BCG strain—the most potent-multi-dose frequency [74]. This makes most observational studies of neonatal BCG for T1D impact inapplicable unless bolstered by biomarker changes [30].

Our hypothesis before launching clinical trials with BCG was that humans would display pancreas recovery or regeneration, based on the results with the NOD model [75]. Instead, in type 1 diabetics with early age of onset, there was no evidence of significant pancreas recovery in our trials of adults with early-onset type 1 diabetes despite consistent evidence of long-term HbA1c improvement (Table 1). In contrast, the very active autoimmunity in the pancreas of LADA diabetics, a disease state with much more remaining baseline pancreas function, showed disease halt and recovery of stimulated C-peptide. Of course these long-term type 1 diabetic subjects had been diabetic since childhood, averaging over 15 years of disease at the trial start and therefore the pancreatic insulin-secreting cells functionally, through glucagon stimulation, were shown not to be present at trial start and also not to be present after BCG immunotherapy but how could the HbA1c stably drop. This paradox shifted our research to non-pancreatic mechanisms.

## 4. BCG’s Mechanisms of Glycemic Improvement in T1D

### BCG Shifts Immunometabolism

All the BCG clinical trials in which C-peptide is measured found no change. Our 20-week Phase 1A trial of BCG showed transient, but not sustained, improvement in C-peptide from negligible baseline levels [64]. The next trial, Phase 1B, showed the same absence of C-peptide recovery through 8 years of follow-up [28] despite significant glycemic improvement to near-normal levels.

The paradox of glycemic improvement without pancreas recovery led to the study of non-pancreatic mechanisms using multiple methods and at multiple levels of analysis: the systemic level (by metabolomics), cellular level (quantitative glucose uptake assay), organ level (longitudinal PET/CT imaging) and gene-level analyses (transcriptional profiling by mRNA-seq; DNA methylation profiling by BeadChip arrays).

Building on the well-known observation that microorganisms reprogram immune cell metabolism [76], Phase 1B evaluated BCG (vs placebo) recipients for metabolic changes by metabolomics and mRNAseq. It found that BCG triggers a shift in T cell (and monocyte) metabolism. The shift was from overreliance on the Krebs cycle and oxidative phosphorylation, a state of low glucose utilization, to aerobic glycolysis, a high glucose-utilization state [28]. Aerobic glycolysis (also known as the Warburg effect) refers to rapid breakdown of glucose for ATP even in the presence of oxygen and is commonly used by activated immune cells to rapidly proliferate.

In the search for evidence, the Phase 1B trial analyzed 690 metabolites in blood over time. This RCT reported higher levels of glucose processing metabolites in BCG recipients, consistent with greater uptake and utilization of blood glucose. This included greater levels of purine synthesis intermediates. In gene expression assays, the trial found upregulated expression of proteins controlling the pentose phosphate shunt, a pathway that branches off early in glycolysis to supply ribose-5-phosphate for biosynthesis of purines. Aerobic glycolysis, unlike oxidative phosphorylation, drives increased flux through the pentose phosphate shunt, thereby enhancing purine biosynthesis needed for immune cell activation and rapid proliferation.

The mRNAseq analysis further supported the systemic shift to aerobic glycolysis seen by metabolomics. At baseline, T1D lymphocytes upregulated OXPHOS-related genes and downregulated glycolysis-related genes. The BCG group (vs placebo) showed upregulated expression of glucose transporters and enzymes involved in early glycolysis (HK2, PFKFB3) while it downregulated expression of late glycolysis enzymes. This suggests that BCG does not simply “turn on” all of glycolysis intermediates uniformly, but instead selectively accelerates regulated glucose entry into the lymphoid system. The final piece of the puzzle was provided by the metabolomic finding that BCG recipients had higher serum lactate and pyruvate, classic systemic indicators of a metabolic shift toward aerobic glycolysis.

Three subsequent studies confirmed or expanded understanding of the shift to aerobic glycolysis. A 2-year open-label trial of BCG recipients [30] reproduced the finding of increased cellular uptake of glucose using another BCG strain (Tokyo vs. earlier Sanofi strain) and quantified the extent of uptake by an in vitro glucose uptake and utilization assay, 2-NBDG, a fluorescently tagged glucose analog. Uptake also reproduced the magnitude and time course of the HbA1c reduction found in the Phase 1B trial. While studying cells from a separate group with LADA, no baseline monocyte or lymphocyte glucose uptake defect was found by the quantitative 2-NBDG assay. The divergence between T1D and LADA was reproduced later in vivo by the 5-year Phase 2 RCT: LADA participants showed no HbA1c reduction over a 5-year observation time after BCG, whereas T1D participants did [65,66].

Using mRNAseq, we showed in CD4 T cells and monocytes from T1Ds studied over 56 weeks that BCG upregulated transcription of mTOR. MYC’s upregulation serves as a central switch for improved glucose metabolism, considering that BCG also activated nearly two dozen MYC-target genes, affecting four metabolic pathways, including aerobic glycolysis, that accelerate utilization of extracellular glucose [27]. The four pathways supply energy to support immune cell proliferation and activation. Enhanced proliferation and activation would also represent a way of amplifying the metabolic reprogramming in daughter cells.

The role of the spleen. All findings of a BCG-induced shift away from OXPHOS, towards the high glucose utilization state of aerobic glycolysis, culminated in a 3-year open-label trial of BCG’s effects on glucose regulation with longitudinal PET/CT imaging [24]. The PET/CT imaging was designed to longitudinally map organ-specific changes in uptake of the glucose analog, 18F-fluorodeoxyglucose (18F-FDG). The trial lacked a placebo group, because exposing a placebo group to repeated imaging procedures and offering no potential benefit would have been unethical. Like all prior long-term trials, the study by Dias and colleagues [24] found a significant drop in HbA1c over time.

The trial found that the greatest increase in 18F-FDG uptake relative to baseline occurred in the spleen. At year 2, the splenic standardized uptake value ratio (SUVR) increased by 47% from baseline, whereas no other organ demonstrated a significant increase over time, nor one even close to the same magnitude. This marked increase in splenic glucose uptake coincided with the onset of HbA1c reduction. The investigators also conducted an experimental study in BALB/c mice by administering BCG and assessing bacterial distribution through colony detection. BCG colonies were most abundant in the spleen, with lower levels detected in the liver and bone marrow. Based on these findings, the authors concluded that the spleen, with its large population of lymphocytes and monocytes, becomes a major bodily site through which BCG mediates its glucose-lowering effects in T1D.

The potential role of the spleen in glucose regulation is further supported by observational studies reporting long-term diabetic-related outcomes after surgery removing the spleen. Compared with matched controls, splenectomy patients exhibit, years later, persistently higher blood glucose levels [77] and a 2.0-fold to 2.35-fold higher risk of incident diabetes, according to two nationwide population-based cohort studies involving thousands of participants [78,79].

## 5. Immunometabolic Shift Driven by Epigenetic Modulation

The evidence from our human trials collectively shows that BCG gradually shifts immunometabolism from overreliance on OXPHOS toward aerobic glycolysis. By 3 years after the first dose, the spleen becomes a bodily site for the BCG-driven shift to aerobic glycolysis [24]. The spleen’s large volume of lymphocytes could explain systemic reduction in HbA1c. That begs the question: what controls the shift to aerobic glycolysis?

The key to what is happening upstream was provided by epigenetic analysis [25]. Epigenetics was a reasonable upstream trigger to study in the BCG causal chain, considering that bacteria have a known capacity to epigenetically modify host DNA [80,81]. Epigenetics (via DNA methylation changes) is a durable process and heritable through cell division. Epigenetics potentially provides a mechanism by which limited BCG dosing could induce the kinds of long-term and durable immune system shifts we have observed.

Dias and colleagues [24] thus sought to determine whether epigenetics plays a role in lymphoid metabolic reprogramming. Using a methylation bead chip on genomic DNA from CD4+ T cells, the study found, at baseline, that T1Ds overexpressed two critically important genes that directly or indirectly repressed glycolysis. Both genes had promoter CpG sites hypomethylated (overexpressed) relative to controls.

The first was a gene, KDM2B, that modifies histones and represses gene function. This gene harbored CpG sites earlier found to have been abnormally methylated in a type 2 diabetes epigenome-wide association study. In T1Ds, BCG was found over a 3-year period to gradually re-methylate KDM2B, thereby diminishing its expression. By mRNAseq, the team confirmed increasingly less mRNA transcription over time. The repression or silencing of a gene for a histone demethylase has context-dependent effects, based on cell type among other factors [82]. In the context of activated immune cells in T1D, KDM2B silencing is thought to shift chromatin to a more transcriptionally active state. That, in turn, is thought to increase glycolysis, perhaps by an earlier, BCG-driven and MYC-activated, metabolic program. Altogether, KDM2B repression was induced by BCG’s upstream epigenetic modulation exerted at the DNA level, but the functional consequence occurs via histone modulation.

The other gene of interest, DDIT4, was also hypomethylated at baseline at CpG sites in T1D CD4 T cells compared to controls [25]. Using the same methodology, CpG sites were found to be remethylated by BCG exposure. Re-methylation of this gene reduces its expression, as confirmed by mRNAseq. Because DDIT4 is a well-known inhibitor of the regulatory enzyme mTOR, gene repression increases DDIT4 activity. mTOR controls glucose uptake, glycolysis, and other cellular functions tied to growth.

Higher mTOR activity is known to promote MYC expression and function primarily by boosting the translation efficiency of MYC mRNA into protein through the mTORC1 pathway and by enhancing MYC stability via mTORC2 signaling [83]. While neither this BCG study nor that of the MYC study [27] directly demonstrates the signaling cascade triggered by BCG-driven epigenetics, the results are biologically consistent with a model in which BCG enhances long-term mTOR signaling which, in turn, supports MYC-driven metabolic reprogramming of immune cells.

## 6. BCG Induces Immunosuppressive Tregs

Regulatory T cells (Tregs) are immunosuppressive cells that constitute about 5–10% of peripheral CD4+ T cells. Tregs maintain immune self-tolerance and immune homeostasis. They express the lineage-defining transcription factor FOXP3, high levels of CD25 (IL-2 receptor α chain), and constitutive CTLA-4, among other key markers [84]. There is strong evidence that Tregs protect pancreatic β-islet cells through multiple mechanisms: by suppression of insulin-autoreactive effector T cells, modulation of antigen-presenting cell function, inhibition of pro-inflammatory cytokine production, and limitation of islet inflammation, among others. Impaired Treg function—and, in some individuals, reduced Treg number or activation state—contributes to the breakdown of immune tolerance and the pathogenesis of T1D [29,85,86].

Our Phase 1A trial showed BCG treatment, on an acute basis, increased the numbers of circulating Tregs in BCG-treated subjects vs. paired healthy controls, while the placebo-treated subjects remained stable. Over the course of the 20-week trial, the BCG group had a higher area under the curve, measured by cumulative Treg ratios (patients to controls) [64]. BCG’s acute impact on Tregs is mediated by its induction of tumor necrosis factor (TNF) release by infected monocytes and other innate immune cells. TNF, in turn, induces Tregs by signaling through their tumor necrosis factor receptor 2 (TNFR2), which is highly expressed on potent Tregs. The combination of Treg induction, along with selective elimination of most autoreactive T cells (also by TNF induction), led to a transient restoration of some endogenous insulin production. But this transient improvement was not sufficient to translate into an HbA1c decrease, as described earlier.

The lengthier study of Treg induction by BCG was part of a broader epigenetic analysis [26] discussed in the next section. What’s noteworthy here is that the study provided a longitudinal analysis over a 3-year period. It reported on baseline methylation defects in Tregs, followed by BCG’s induction of Tregs (in relation to HbA1c levels). At baseline, T1Ds had over-methylation of the FOXP3 gene. This finding suggested an epigenetic barrier to induction of stable Tregs. BCG immunotherapy was associated with gradual demethylation of Treg signature genes starting from around year 1. The pace of BCG-induced demethylation of the Foxp3 gene was faster during years 1–2 in early-onset T1Ds (<20 years) than later-onset (>20 years), roughly corresponding to T1D vs. LADA. By year 3, however, both autoimmune diabetes groups had achieved equal levels of demethylation.

Tregs in autoimmune diabetes are more likely to be immature CD45RA^+^ Tregs, meaning that they have a resting or naïve phenotype [87]. In in vitro suppression assays, CD45RA^+^ Tregs are less suppressive than CD45RO^+^ Tregs. BCG therapy is associated with early demethylation of the CD45 gene at year 1, after which demethylation slowly increases to year 3 [26]. Demethylation is correlated in time with the finding that CD45RA naïve Tregs transition to CD45RO mature Tregs, according to longitudinal flow cytometry and antibody phenotyping. The CD45RA marker on Tregs, assessed by mean fluorescent intensity, is gradually lost by the BCG group, while the same marker in placebo (not BCG treated subjects) recipients does not decrease over time. In summary, BCG recipients show Treg expansion in vivo over 1–3 years based on flow cytometry, and the expansion correlates with the timing of glycemic improvement by HbA1c reduction [26].

## 7. BCG-Driven Epigenetic Modulation of Tregs

The 8-year Phase 1B trial included a study of whether BCG could durably reset Tregs by assessing the DNA methylation status of six Treg signature genes using Bead Chip technology. As early as week 8 after BCG exposure, dozens of CpG sites within these genes exhibited significant DNA demethylation compared with baseline. Because DNA demethylation is expected to increase gene transcription, the study found upregulated mRNA expression of the same Treg signature genes, as measured by mRNAseq [28]. The question is whether this demethylation is part of an early, TNF-driven Treg induction through short-term acute immune memory, or is this a long-term, durable form of Treg induction?

Long-term Treg methylation changes were studied in a 3-year, longitudinal, open-label study of BCG for longstanding autoimmune diabetes [26]. The focus of this epigenetic study was on the subset of Tregs known to be highly potent in their suppression capacity. Using an EPIC Bead Chip which interrogates 850,000 CpG sites across the genome, the study examined methylation changes across 11 Treg signature genes, as well as more detailed analysis of the FOXP3 gene locus which contains its TSDR region, a regulatory element that serves as the best marker of stable Treg commitment to the suppressor lineage. The 11 signature genes for highly potent Tregs under study were: Foxp3, TNFRSF18 (GITR), CD25, IKZF2 (Helios), IKZF4 (Eos), CTLA4, TNFR2, CD62L, Fas, CD45, and IL2. The inclusion of transcription factors of the Ikaros family (Helios and Eos) reflects their crucial role in stabilizing Foxp3 protein expression and in ensuring Treg suppressive function.

The main finding was that BCG induced a gradual, 3-year process of progressive demethylation of nine of the 11 Treg signature genes at the majority of their CpG sites. At baseline, the Foxp3 gene, in particular, was significantly overmethylated relative to non-diabetic controls. Three years later, 17 of its 22 CpG sites became demethylated, including the TSDR region. Over the three years, the demethylated Treg genes showed increased transcription at similar serial time points. The genes that did not show demethylation did not show mRNA changes. Separate evaluation was made of the TSDR site. By 58 weeks, there was a pattern of increasing demethylation. At the 58-week mark, the average Treg population, assayed by flow cytometry, was increased in relation to baseline. The study concluded that multi-dose BCG therapy triggered long-term induction of potent Tregs through epigenetic demethylation of Treg signature genes. The time course correlated with that of BCG’s glycemic improvement.

Another reason for the paucity of Tregs in T1D may trace to another set of genes found to be hypermethylated. The problem was discovered at baseline in a BCG trial when participants’ T cells were found to exhibit a quantitative defect in T cell receptor (TCR) density indicative of reduced expression vs. non-diabetic controls [31]. The reduction was identified to be the product of hypermethylation of CpG sites on T cell Cell Receptor (TCR)-related genes, via analysis by EPIC BeadChip. Ninety of the 200 genes most hypermethylated were found to be TCR-related. The study ruled out recombination of the TCR sequences. BCG treatment, over three years, demethylated the genes of interest. The functional consequence—improvement of signal strength through restoration of TCR density—was assessed by protein array showing increased phosphorylation of multiple kinases in the TCR signaling pathway. Improved signal strength has the potential to resume signaling that would trigger Treg proliferation, but this was not evaluated.

Taken together, these studies reveal that epigenetic reprogramming of Treg signature genes or restoration of TCR density may contribute to BCG’s induction of potent Tregs. Normalization of Tregs has potential to restore their cellular functions (and/or numbers) related to controlling autoimmunity and inflammation. This, in turn, may help to explain the Phase 2 results regarding insulin dose reduction and HbA1c reduction in T1Ds. LADA responds to BCG by preservation of pancreatic function, which likely results from attenuation of autoimmunity. Restoration of these functions is also suggestive of greater Treg activity: less insulin resistance and fewer autoantibodies. The induction of Tregs with BCG immunotherapy in LADA is probably the reason this study’s data also shows multi-dose BCG removes insulin resistance by the systemic removal of inflammation, a known cause.

## 8. Conclusions and Implications for T1D Treatment and Prevention

The body of six clinical trials is consistent and reproducible: a few doses of BCG immunotherapy for autoimmune diabetes has multiple advantages for treatment: (1) lack of systemic moderate or severe adverse events; (2) glycemic efficacy in an adult population with longstanding T1D, the population most often encountered clinically yet rarely included in clinical trials; (3) insulin dose reductions; (4) protection from insulin resistance and autoimmunity attenuation for LADA apart from glycemic improvement; (5) years-long impact without chronic dosing; and (6) affordability.

The evidence of BCG’s mechanism of action helps to explain how the causal chain may begin at the level of epigenetic modulation of DNA, a mechanism capable of long-term stability of outcomes, without altering sequences; how BCG reprograms immune cell metabolism and Treg activation; and why BCG takes up to 3 years to realize the benefits. The shift in immunometabolism helps to explain how BCG can work in adults without a functioning pancreas, one that is ravaged by autoimmunity and no longer capable of producing any insulin.

Since BCG is not an approved vaccine in the US and is in short supply globally, evidence in international settings now confirms the ability of BCG to lower blood sugars, reduce HbA1c levels and also lower autoantibodies. Using neonatal BCG vaccines or no BCG vaccines at birth as the stratification criteria, only the BCG-vaccinated adults with type 1 diabetes had lowered HbA1c, lowered IDAA1c, as well as the activation of Tregs with the expected metabolic increases in glycolysis metabolites [68]. Similarly, adult-administered BCG to type 1 diabetic subjects improved glycemic control and reduced autoantibodies measured for 18 months. Both HbA1c and autoantibody levels decreased, supporting the therapeutic promise of BCG in existing diabetes [88]. There is also some retrospective evidence supporting BCG as a preventive intervention that delays onset or reduces incidence of T1D with three or more vaccines [69,70,89]. The evidence of safety, treatment benefits, risk modification, and potential prevention suggests that BCG, through unique metabolic and Treg mechanisms, improves diabetes care and control, not achievable with the standard of care.

## Figures and Tables

**Table 1:** Human BCG clinical trials of glycemic improvement in autoimmune diabetes.

**Trial Description/Cohort**	**Proof of Concept Phase IA Adult Clinical Trial in T1D with BCG**	**Phase IB Adult Clinical Trial in T1Ds with BCG**	**Initial Phase II Open-Label BCG Trial in T1D (Subset of Phase II Study)**	**Initial Phase II Open-Label BCG T1D LADA Trial (Subset of Phase II Study)**	**Phase II Adult BCG Clinical Trial in T1D PET Cohort**	**Phase II T1D BCG—Infections**	**Phase III T1D BCG—Infections**
IND #	10435	016434	016434	016434	016434	016434	016434
IRB #	2007P001347	2012P002243	2013P002633	2013P002633	2013P002633	2020P001462	2020P001462
Sample Size	*n* = 6, *n* = 42 reference placebos T1D	*n* = 6, *n* = 42 reference placebos	*n* = 3 T1D	*n* = 3 T1D	*n* = 6 T1D	*n* = ~150 T1D	*n* = ~150 T1D
Trial Duration	22 Weeks	8 Years	5 Years	5 Years	5 Years	18 months	24 months
Trial Design	RCT	RCT/Open Label after year 05	Open Label	Open Label	Open Label	RCT	RCT
Trial Stage	Phase 1A	Phase 1B	Phase 2	Phase 2	Phase 2	Phase 2	Phase 3
Outcome	Acute death of auto-reactive T cells, C-peptide increase, Treg induction	Long-term decrease in HbA1c even with longstanding T1D with no pancreatic function	Long-term decrease in HbA1c observed	LADA: no change in HbA1c but halt of insulin resistance and autoimmunity	Spleen takes up sugar; long-term drop in HbA1c	Protection from COVID and infections	Protection from COVID and infections
Publications	PlosOne2012 [64]	Nature Vaccines [28]	Cell iScience 2021 [30]	Diabetes [65]	Nature Scientific Reports [24]	Cell Report Medicine [5]	Cell iScience [6]
**Trial Description/Cohort**	**Phase II Adult BCG Clinical Trial: Juvenile Onset (T1D)**	**Phase II Adult BCG Clinical Trial in adult-onset T1D (LADA)**	**Phase II Adult Clinical Trial: Expanded T1D Cohort**	**Phase II Secondary Use Authorization: Alzheimer’s disease in T1D**	**Phase II Adult Clinical Trial: Long-term Follow-Up of T1D**	**Phase II Pediatric Clinical Trial: Adolescent Onset T1D Cohort**	**Phase II Pediatric Clinical Trial: Recent Onset T1D Cohort**
IND #	016434	016434	016434	016434	016434	016434	016434
IRB #	2013P002633	2013P002633	2013P002633	2025P002874	2020P003578	2019P002835	2019P002835
Sample Size	*n* = ~50 T1D	*n* = ~100 T1D	*n* = ~32 T1D	*n* = ~150 T1D	*n* = ~150 T1D	*n* = ~150 T1D	*n* = ~100 T1D
Trial Duration	5–10 Years	5–10 Years	5–10 Years	5–10 Years (Ongoing *)	5–10 Years (Ongoing *)	10 Years (Ongoing *)	5–10 Years (Ongoing *)
Trial Design	RCT	RCT	Open Label	RCT	Follow-Up	RCT	RCT
Trial Stage	Phase 2	Phase 2	Phase 2	Phase 2	Phase 2	Phase 3	Phase 3
Outcome	Long-term drop in HbA1c, decrease in insulin use, restored time with normoglycemia	Restored peak C-peptide, halt of autoimmunity, prevention of insulin resistance	Ongoing	Correction of FDA-approved biomarkers—pTau217, Amyloid beta showing prevention of AD	Fully enrolled, ongoing	Fully enrolled, ongoing	Fully enrolled, ongoing
Publications	Diabetes [65]	Diabetes [65]	Ongoing	CTAD 2025 [11]	Diabetes [65]	Ongoing	Ongoing

## Data Availability

No new data were created or analyzed in this study. Data sharing is not applicable to this article.
